# Poly(ethylene glycol)-*b*-poly(1,3-trimethylene carbonate) Amphiphilic Copolymers for Long-Acting Injectables: Synthesis, Non-Acylating Performance and In Vivo Degradation

**DOI:** 10.3390/molecules26051438

**Published:** 2021-03-06

**Authors:** Silvio Curia, Feifei Ng, Marie-Emérentienne Cagnon, Victor Nicoulin, Adolfo Lopez-Noriega

**Affiliations:** MedinCell S.A., 3 Rue des Fréres Lumiére, 34830 Jacou, France; silvio.curia@medincell.com (S.C.); feifei.ng@medincell.com (F.N.); marie.cagnon@medincell.com (M.-E.C.); victor.nicoulin@gmail.com (V.N.)

**Keywords:** long acting injectables, aliphatic polycarbonates, acylation, polyesters, poly(lactic acid), amphiphilic copolymers, octreotide, liothyronine

## Abstract

This article presents the evaluation of diblock and triblock poly(ethylene glycol)-*b*-poly(1,3-trimethylene carbonate) amphiphilic copolymers (PEG-PTMCs) as excipients for the formulation of long-acting injectables (LAIs). Copolymers were successfully synthesised through bulk ring-opening polymerisation. The concomitant formation of PTMC homopolymer could not be avoided irrespective of the catalyst amount, but the by-product could easily be removed by gel chromatography. Pure PEG-PTMCs undergo faster erosion in vivo than their corresponding homopolymer. Furthermore, these copolymers show outstanding stability compared to their polyester analogues when formulated with amine-containing reactive drugs, which makes them particularly suitable as LAIs for the sustained release of drugs susceptible to acylation.

## 1. Introduction

Long acting injectables (LAIs) have achieved wide success due to their numerous clinical benefits over oral therapies, such as enhanced adherence to treatment, increased bioavailability for some drugs and avoidance of first-pass metabolism [1,2,3,4,5]. For these reasons, in the last decades this therapeutic route has become increasingly ubiquitous in the cure of chronic health conditions and long-term illnesses [6,7,8]. Additionally, differently from traditional non-degradable releasing implants, bioresorbable LAIs do not require surgical removal once the medical treatment is completed. Hence, their usage has been shown to have a significant positive impact on the quality of life of patients throughout the cure process, avoiding the uncomfortable need for surgeries and excisions [6].

Several technologies and approaches can be utilised to obtain LAIs. For instance, encapsulation of the API into polymeric microspheres or formation of poorly soluble salts of the active pharmaceutical ingredient (API), to name just a few [8,9,10]. An alternative system is represented by in situ forming depots (ISFDs), which are also referred to as in situ forming implants (ISFI). With this approach, the active molecule is solubilised or suspended into a solution containing an appropriate resorbable polymer, or a blend of polymers [6]. In some cases, a suitable biocompatible organic solvent, such as dimethyl sulfoxide (DMSO) [9,10,11,12], is used to solubilise the polymers. Upon injection and contact with an aqueous environment, the solvent diffuses out, and the polymer, purposely designed to be insoluble in water under physiological conditions, precipitates. This process results in the physical entrapment of the API. Henceforth, the drug release is controlled by diffusion and polymer degradation [6].

Typically, polyesters like poly(lactide) (PLA) and copolyesters prepared by using lactide (LA) in combination with other monomers, such as glycolide (GA) in poly(lactide-*co*-glycolide) (PLGA), are employed for this kind of technology [13]. Similarly, the utilisation of amphiphilic block copolymers composed of a chain of poly(ethylene glycol) (PEG) covalently connected to one or more polyester chains has also been widely investigated [14,15,16]. For instance, at MedinCell we have successfully employed a combination of amphiphilic diblock (DB) and triblock (TB) PEG-PLA copolymers to obtain ISFDs with tuneable and controlled drug loading and release [6,17].

Nonetheless, it is well known that some amine-containing drug molecules, such as proteins, hormones and peptides, are not entirely compatible with the acidic by-products of polyesters [18,19,20,21]. For instance, LA- and GA-containing polymers are known to be generally unsuitable for the sustained delivery of peptides due to acylation side-reactions leading to generation of impurities of these active molecules [22,23,24]. In more detail, in formulations including PLAs or PLGAs, nucleophilic chemical groups such as the amino moieties have been shown to be the major targets for acylation, as a result of their interaction with lactic and glycolic acid species ensuing from the hydrolytic scission of the ester linkages along the polymeric backbone [23,24]. Additionally, not only can some of the impurities that are produced following acylation result in a decreased efficacy of the drug products, but also further concerns linked to safety and potential toxicity might arise [25,26,27].

In the past few decades, aliphatic polycarbonates have been widely investigated and these polymers have attracted significant interest as materials for biomedical applications owing to their biocompatibility and to their peculiar in vivo degradability [28,29,30,31]. Poly(trimethylene carbonate)s (PTMCs) and the related PEG-PTMC copolymers can be prepared from the ring-opening polymerisation (ROP) of the cyclic monomer trimethylene carbonate (TMC), using conventional organometallic catalysts, organic catalysts and enzymes [28,31,32,33,34,35]. PTMC is a hydrophobic amorphous polymer, with glass transition temperature (*T_g_*) in the −20 °C range, which can be suitable as a hydrophobic block in amphiphilic copolymers or as a soft material for scaffold applications [32,34,36,37]. In contrast to traditional polyesters commonly used for biomedical applications, PTMC is resistant to non-enzymatic hydrolysis [31,38]. Indeed, it has been demonstrated that the degradation of PTMCs proceeds with a surface erosion mechanism due to enzymatic degradation. This was confirmed by the decreased size and mass accompanied by a constant molecular weight observed for PTMC implants over time, and by the lack of degradation in vitro in the absence of degrading enzymes (e.g., lipases) [36,38,39]. Additionally, detrimental acidic products are not released upon degradation of PTMC, thus opening novel opportunities for the sustained delivery of APIs typically incompatible with acidic environments [28,30,32,33,34,35,37,39,40].

From this knowledge, we hypothesised that PTMC-based copolymers could be viable bioresorbable materials beneficial to overcome acylation issues and expand the resource base of polymeric materials suitable for LAI applications. With this goal, three PTMC-based polymers were synthesised, and their respective in vivo degradation behaviour was examined. In more detail, one DB PEG-*b*-PTMC copolymer, one TB PTMC-*b*-PEG-*b*-PTMC copolymer and one PTMC homopolymer, with molar mass comparable to that of the PTMC segments composing the hydrophobic portions of the two block copolymers, were investigated. The overall aim was to highlight the effects of the structural features on the degradation behaviour and, particularly, the influence of the presence of the hydrophilic PEG segment.

The three polymers were synthesised via tin-catalysed ROP of TMC utilising methoxy PEG (mPEG), PEG and 1-octanol as initiators (for the DB, TB and homopolymer, respectively). Silica gel flash chromatography was employed in order to remove undesired side-products and ultimately obtain purer copolymers to be tested and carefully compared with their related PTMC homopolymer. This allowed for a clearer assessment of the behaviour associated to pure TB PEG-PTMC, DB mPEG-PTMC and PTMC homopolymer during the in vivo studies, thus ensuring a more precise structure-property-degradation evaluation.

Furthermore, injectable formulations with octreotide and liothyronine, a peptide and a hormone, have been prepared to evaluate the usefulness of PEG-PTMCs for the preparation of pharmaceutical compositions containing APIs susceptible to acylation [21,41,42]. The stability of these formulations was compared to that of reference compositions prepared with a TB PEG-PLA possessing chemical and structural features comparable to those of the synthesised amphiphilic TB PEG-PTMC.

Overall, the results of this study convey a fundamental understanding towards the coherent design of novel LAIs, expanding the current knowledge on the distinctive in vivo resorption of PTMC-based soft implants, and providing useful information for the development of effective systems for the sustained release of therapeutic molecules characteristically incompatible with polyester-containing delivery systems.

## 2. Experimental

### 2.1. Materials

Tin(II) 2-ethylhexanoate (Sn(Oct)_2_, 92.5–100%) and 1-octanol (≥99.0%) were purchased from Sigma Aldrich (Saint-Louis, MO, USA) and used without further purification; the catalyst was stored at 4 °C, while the alcohol was kept in the dark at room temperature (RT). Poly(ethylene glycol) 1000 g mol^−1^ (PEG, >99.0%) and methoxy poly(ethylene glycol) 350 g mol^−1^ (mPEG, >99.5%) were purchased from Clariant (Muttenz, Switzerland) and used without further treatment and stored at −20 °C. Trimethylene carbonate (TMC, >99%) was purchased from Foryou Medical (Huizhou, China), used as received and stored under vacuum at −20 °C. The PEG-PLA polymer (*M_n_* = 9.0 kg mol^−1^, Ð = 1.6) used for comparison in the drug recovery studies was purchased from Corbion (Diemen, Netherlands), stored at −20 °C and used as received. The two APIs, octreotide acetate (>99.0%) and liothyronine sodium (>99.9%), were purchased from Bachem (Bubendorf, Switzerland) and Azico Biophore (Lankelapalem, India), respectively. Both molecules were stored at −20 °C and used as received.

Before use, all the chemicals stored at low temperatures were allowed to thermalise to RT in order to minimise environmental water intake. The water content of the (m)PEG macroinitiators was routinely tested via Karl Fischer analyses using a V30 Volumetric Titrator (Mettler Toledo, Greifensee, Switzerland) to ensure an appropriately low water content (<1 wt.%).

All the used solvents were of certified reagent grade or high-purity liquid chromatography grade, purchased from either Honeywell (Charlotte, NC, USA) or Carlo Erba (Milano, Italy) and used as received. Silica gel technical grade (pore size 60 Å, 230–400 mesh, 40–63 μm particle size) was purchased from Sigma Aldrich (Saint-Louis, MO, USA) and used as received. Nitrogen (N_2_, ≥99.999%) was produced via an Air Liquide (Paris, France) N_2_ generation system.

### 2.2. Methods

#### 2.2.1. Synthesis of (m)PEG-PTMC Block Copolymers and PTMC Homopolymer

**Scheme 1:**
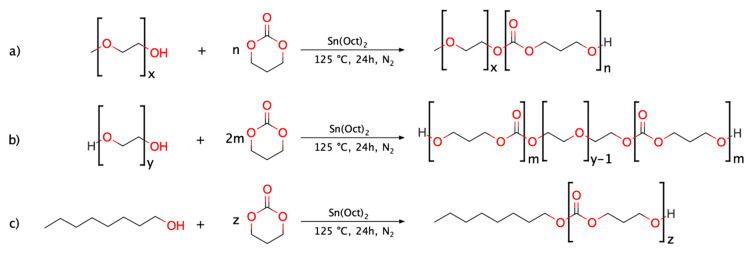
Synthesis of PTMC-based polymers: (**a**) DB mPEG-PTMC; (**b**) TB PEG-PTMC and (**c**) PTMC homopolymer.

The reactions were performed following a solvent-free procedure with an overhead Hei-TORQUE Value 100 mechanical stirrer (Heidolph, Schwabach, Germany) set to 30 rpm at each step. The amounts of reactants were calculated so that the total mass of polymer to be produced in each polymerisation reaction was 50 g. In more detail, a 250 mL 3-neck round-bottom flask was dried at 140 °C overnight. Then, the appropriate amount of initiator (namely, mPEG, PEG or 1-octanol, Scheme 1), calculated based on the targeted molecular weight value (i.e., 3.6 kg mol^−1^ for the DB copolymer, 10.3 kg mol^−1^ for the TB and 4.6 kg mol^−1^ for the PTMC homopolymer), was introduced at 80 °C under inert atmosphere (N_2_) whilst stirring. Three vacuum/N_2_ cycles (1 min each) were performed to remove water residues, after which the system was stirred for 20 min under vacuum. Then, the flask was filled with N_2_ and the organometallic catalyst (1 mol% relative to the amount of initiator, in the case of 1-octanol and mPEG, and 2 mol% for PEG) was added and left to stir for activation for 5 min. Finally, TMC was added and the reaction mixture was homogenised for 2 min at 80 °C. Then, the temperature of the oil bath was increased to 125 °C.

The beginning of the reaction (*t_0_*) was taken as the time when the hotplate reached the temperature setpoint (typically in 20–25 min). The reactions were carried out under controlled atmosphere (N_2_) whilst stirring continuously.

In the case of the polymer initiated with 1-octanol (i.e., synthesis of PTMC homopolymer, Scheme 1c), aliquots were collected at specific times (0.5, 1, 2, 3, 21 and 24 h) to monitor the evolution of the polymerisation.

After 24 h, and upon cooling to RT, the polymers were dissolved in acetone (1 g mL^−1^) and a 4-fold excess of ethanol (EtOH) was slowly added directly to the flask to induce precipitation, whilst stirring at 200 rpm. The precipitated polymer was left to settle for 30 min at RT and the top liquid phase containing the monomers and lower molecular weight species was discarded. This procedure was performed twice to ensure a thorough removal of the monomer residues, and the second time the polymers were left to settle overnight at −20 °C to maximise product retrieval.

The catalyst residues were removed via filtration on active charcoal (3M, ZETA PLUS^TM^, 5 × 0.6 cm, 2.8 g), using a Vantage 3000 peristaltic pump (Verderflex, Castleford, UK) set to 20 rpm for 6 h. The residual tin content was less than 20 ppm for all polymers (determined via inductively coupled plasma atomic emission spectroscopy [ICP-AES] by an external contract research organisation [Laboratoire LACAPA, Toulouges, France]).

After purification, the polymers were dried for three days at 10^−2^ mbar at 40 °C.

For increased clarity, the nomenclature used throughout the paper is summarised in Table 1.

#### 2.2.2. Polymer Purification via Flash Column Chromatography (Removal of PTMC Side-Product)

Silica gel column flash chromatography was performed to remove the PTMC homopolymer by-product formed during the syntheses from the two copolymers, in order to obtain purer block copolymers to be used for the in vivo studies. The column (40 cm, 5 cm ∅) was packed by adding a slurry of silica in tetrahydrofuran (THF):heptane 80:20 (eluent 1). Dry loading of the samples was used to optimise separation. Typically, 5 g of polymer product were purified per each column. Eluent 1 was used to remove the PTMC homopolymer, then pure THF (eluent 2) was used to elute the purified copolymers upon the complete removal of the by-product. A manually controlled, positive N_2_ pressure was used to drive the eluents through the stationary phase. The process was monitored through thin layer chromatography (TLC) on ALUGRAM^®^ Xtra SIL G silica plates (Macherey-Nagel, Düren, Germany) using a potassium permanganate (KMnO_4_) staining solution for visualisation. Photos of the TLC plates are available in the Supporting Information (SI). At the end of the separation process, the fractions containing the same species were mixed together, reduced with a rotary evaporator and then thoroughly dried for three days at 10^−2^ mbar at RT.

### 2.3. In Vivo Degradation Study over 3 Months in Rats

The in-life phase was performed at Porsolt (Laval, France) in compliance with the Council Directive No. 2010/63/UE and with the recommendations of the Association for Assessment and Accreditation of Laboratory Animal Care (AAALAC). The study was conducted in 32 male Sprague Dawley rats (Janvier Labs, Laval, France) weighing 280–314 g at the injection day. Animals were randomized in 4 groups of 8 rats corresponding to four testing durations, i.e., 2 weeks (0.5 month), 1 month, 2 months and 3 months.

The formulations, composed by the polymers dissolved in DMSO (Table 2), were administered as a single subcutaneous injection of 160 µL. Dosing was performed in the flank of the animals anaesthetised with isoflurane, using 1 mL Luer Lock syringes equipped with 23G 5/8” needles. In each group, 4 animals received one dose of formulations (a) and (c), respectively in the right and left flank. The 4 other animals received one dose of formulation (b) in the right flank. Overall, each vehicle was injected 16 times in order to obtain quadruplicates at each time point.

According to their group, the animals were euthanised at specific times (0.5, 1, 2 or 3 months) by exposure to carbon dioxide (CO_2_).

Injection sites comprising polymer depots and surrounding biological tissues were collected, stored at −80 °C and shipped to our laboratories for analysis.

#### Depot Recovery and Determination of Weight Loss

Before any treatment, the biological tissues containing the polymer depots were allowed to thermalise to RT for 3 h in sealed containers to minimise environmental water intake. All the excision tools were cleaned with EtOH before use. A photo of each depot was taken immediately upon explantation, after which the depots were introduced into vials of known weight.

The depots were lyophilised, and their dry weight (*W_d_*) was recorded. The depot weight loss (%) over time was determined following Equation (1):

(1) $$\mathrm{Weight}\;\mathrm{Loss}\;\left(\%\right)=\;\frac{W_{i}-W_{d}}{W_{i}}\times 100$$

where *W_i_* is the initial weight of the injected polymer (calculated starting from the specific volume of each injection) and *W_d_* is the weight of the dry depot weight recorded upon lyophilisation.

After weighting, the dry depots were cut in small pieces (2–3 mm) to aid their dissolution in 5 mL of ethyl acetate (EtOAc), leaving them to dissolve overnight on an orbital shaker at RT (90 rpm). The polymer solutions were filtered through 0.45 µm poly(tetrafluoroethylene) (PTFE) filters to exclude traces of undissolved materials (salts, residual biological tissues, etc.). Then, the organic solvent was removed at 10^−2^ mbar at 40 °C. These microfiltered polymers were subsequently used as specimens for the determination of the structural characteristics of the depots.

### 2.4. Stability of Octreotide and Liothyronine in the Presence of TB PEG-PTMC (Acylation Studies)

**Figure 1:**
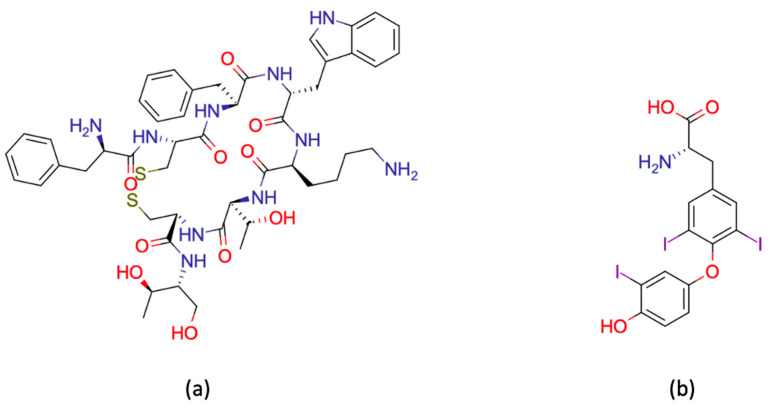
Structures of the APIs used in this study: (**a**) octreotide and (**b**) liothyronine. Both molecules contain primary amino groups susceptible to acylation [24,25,43].

Acylation tests were performed to assess the effects of formulating a long acting injectable based on TB PEG-PTMC copolymer on the stability of two active molecules: octreotide and liothyronine (Figure 1). For comparison purposes, a TB PEG-PLA analogue was also used for the preparation of injectable compositions used as references.

The formulations used for these studies were prepared by adding the required quantity of copolymers and DMSO into glass vials (36.5 wt.% of polymer for the solutions to be used with octreotide and 35.4 wt.% of polymer for the solutions to be used with liothyronine). The vials were then sealed, and the polymers left to dissolve on a roller mixer (60 rpm) at RT until complete dissolution. In parallel, precise amounts of octreotide acetate and liothyronine sodium were weighed in four separate vials and the required amounts of polymer solutions were added to the APIs to obtain final solutions with specific concentrations (Table 3).

The vials were then sealed, agitated using a vortex mixer to yield homogeneous formulations, and kept on a roller mixer (60 rpm) at RT.

#### 2.4.1. Octreotide Acylation Tests

At specific time points, 120 µL of the formulations 1 and 2 were withdrawn and three replicates of approximately 40 µL were weighed into Falcon centrifuge tubes (50 mL). Then, 4 mL of acetonitrile were added to the formulations and the tubes were closed and left to stir on an orbital shaker at RT (90 rpm). The required volume of H_2_O + 0.1% trifluoroacetic acid (TFA) was added to reach a final volume of 20 mL. The tubes were agitated on a vortex mixer and kept at 4 °C until analysis. Before analysis, 1.5 mL of each replicate was transferred into an Eppendorf^®^ tube (1.5 mL) and centrifuged using an Eppendorf Centrifuge 5415 R (5 min, 16,100 RCF). Then, a portion of the supernatant (1 mL) for each sample was collected and transferred into a High-Performance Liquid Chromatography (HPLC) vial. The concentration of octreotide in the formulations at each time was determined by Reverse Phase (RP)-HPLC following the method summarised in the SI.

#### 2.4.2. Liothyronine Acylation Tests

At given timepoints, 510 µL of formulations 3 and 4 were withdrawn and three replicates of approximately 170 µL were weighed into Falcon centrifuge tubes (50 mL). Then, 16 mL of acetonitrile were added to the formulations. The Falcon tubes were then sealed and left to stir on an orbital shaker at RT (90 rpm). The required volume of water was then added to reach the final volume of 20 mL. The Falcon tubes were then closed, agitated on a vortex mixer, and kept at 4 °C until analysis. For the analyses, 1 mL of each replicate was transferred into a HPLC vial. The concentration of liothyronine in each solution was determined by RP-Ultra Performance Liquid Chromatography (UPLC) following the method summarised in the SI.

### 2.5. Characterization

#### 2.5.1. Proton Nuclear Magnetic Resonance (^1^H-NMR)

^1^H-NMR analyses were conducted on a Bruker (Billerica, MA, USA) Avance^TM^ III 300 MHz spectrometer at 25 °C in deuterated chloroform (CDCl_3_) at a concentration 15–20 mg mL^−1^. The number of scans was 32 and the chemical shifts were reported in parts per million (ppm) with respect to the residual solvent peak (7.26 ppm) [44].

For the copolymers, the overall ratio between the number of TMC repeat units and that of ethylene oxide (EO) units in each sample was calculated using the following Formula (2):

(2) $$R=\;\frac{I^{TMC}\left(4.23\;\mathrm{ppm}\right)}{I^{EO}\left(3.65\;\mathrm{ppm}\right)}$$

where *I^TMC^* (at 4.23 ppm) is the integral of the peak attributed to the -CH_2_- adjacent to the carbonate linkages within the TMC units, and *I^EO^* (at 3.65 ppm) is the integral of the resonance attributed to the protons in the EO units of the hydrophilic segment. The spectra of interest are shown in the SI (Figures S1–S3).

#### 2.5.2. Gel Permeation Chromatography (GPC)

The molecular weight distributions of the synthesised samples were analysed using a Malvern Panalytical (Malvern, UK) Viscotek GPCmax separations module with a Viscotek TDA 305 detector (triple detector array). The instrument was equipped with a series of I-MBMMW-3078, I-MBLMW-3078 and I-OLIGO-307 columns kept at 30 °C. The samples were run in tetrahydrofuran (THF) Chromasolv^®^ (15 mg mL^−1^) at a flow rate of 1 mL min^−1^. The system method was routinely calibrated and validated with poly(methyl methacrylate) standards (PolyCAL^®^ TDS-PMMA-NB, Malvern Panalytical).

The refractive index increment (*dn/dc*) of each polymer was determined by injecting six different volumes and plotting the resulting RI signals as a function of concentration. Each polymer was analysed at least twice to ensure data reproducibility. The typical error was less than 5%.

#### 2.5.3. Differential Scanning Calorimetry Analyses (DSC)

Differential scanning calorimetry assays were performed using a Mettler Toledo (Greifensee, Switzerland) DSC3+ calibrated with indium standards. In a typical experiment, the sample (generally 5–10 mg) was weighted into a 40 µL aluminium crucible (Mettler Toledo). This crucible was then capped with the matching lid and sealed using the appropriate press. The sample was cooled down to −80 °C and an initial ramp up to 100 °C was performed to both record the properties of the pristine material and erase its thermal history. The sample was then cooled to −80 °C and a second heating scan up to 200 °C was carried out to determine the thermal transitions. All the scans were performed at a heating rate of 10 °C min^−1^ and the experiments were carried out under a N_2_ flow (50 mL min^−1^). All the heating and cooling ramps were followed by a 10-min isothermal step to equilibrate the sample. The *T_g_* was taken as the temperature at the half-height point of the heat flow change. Each experiment was performed in duplicates to give data confidence. The error on the measured *T_g_*s was typically 0.2–0.5 °C and the average values (rounded to the closest integer) are reported.

### 2.6. HPCL/UPLC Analyses

Liquid chromatography analyses were used to quantify the amount of remaining API (octreotide or liothyronine) at each time point during following the acylation tests. The specific details about the utilised instruments and methods are available in the SI.

## 3. Results and Discussion

### 3.1. Synthesis of (m)PEG-PTMC-Based Copolymers and PTMC Homopolymer

In this paper, TMC was polymerised through ROP in the melt at 125 °C with different initiators to produce two amphiphilic PEG-PTMC copolymers (DB and TB, respectively), and one PTMC homopolymer. The yield for each reaction was typically higher than 80%.

**Figure 2:**
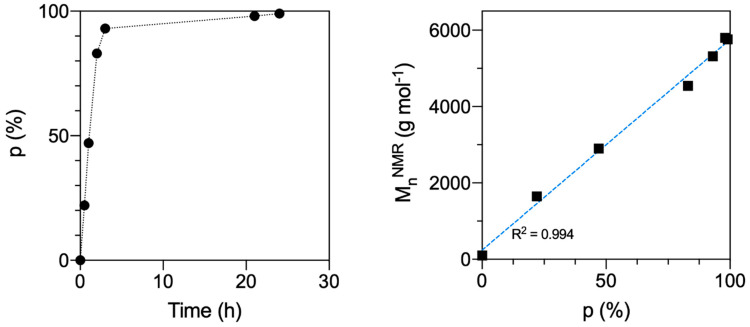
Plots of TMC conversion calculated from ^1^H-NMR as a function of time (**left**), and M_n_^NMR^ as a function of conversion (**right**) for the synthesis of the PTMC homopolymer. On the left, in the p (%) vs. time (h) plot the dotted black line is included as a guide to the eye. The M_n_^NMR^ (g mol^−1^) vs. p (%) data points were fitted with a linear equation with very high correlation (dashed blue line, R^2^ = 0.994). The error was typically less than 5% and, thus, the data point symbols are bigger than the error bars. Overall, the kinetic study confirmed the achievement of a high monomer conversion and a good polymerisation control at the reaction conditions.

As a preliminary investigation, a kinetic study was conducted on the PTMC homopolymer to ensure the obtainment of high monomer conversion at the selected reaction conditions. To this end, ^1^H-NMR analyses were used to calculate the TMC conversion at each time, by comparing the monomer peak at 4.45 ppm with that assigned to the protons adjacent to the carbonate linkages in the polymer at 4.23 ppm. The resulting graph of monomer conversion (*p*) as a function of time shows that an almost quantitative conversion is obtained after approximately 24 h (Figure 2). Additionally, the plot showing the number average molecular weight (*M_n_^NMR^*) against conversion confirms that a controlled chain growth polymerisation is achieved, since the *M_n_* value is a linear function of monomer conversion [45].

Hence, accordingly with these results, all the amphiphilic copolymers were synthesised using the same conditions and with equal reaction duration of 24 h. For these copolymers, the obtainment of products with the targeted characteristics and high monomer conversion was confirmed through ^1^H-NMR at the end of the reactions. The spectra of the polymers after precipitation in EtOH are shown in the SI.

### 3.2. Purification of (m)PEG-PTMC-Based Copolymers via Column Flash Chromatography

In order to have a clear distinction between the behaviour associated to pure TB PEG-PTMC, DB mPEG-PTMC and PTMC homopolymer during the in vivo degradation study, the relative purity of the copolymers had to be carefully scrutinised. As a matter of fact, the obtainment of PTMC homopolymer by-product during the syntheses of the block copolymers was confirmed by preliminary TLC analyses. The presence of this side-product was detected regardless of the reaction conditions and drying protocol applied for the reactants.

Nevertheless, the PTMC by-product present in the two copolymers was effectively removed via column flash chromatography utilising two eluents (eluent 1 THF:heptane 80:20 to remove the by-product fractions, and eluent 2 THF 100% to retrieve the purified copolymers).

**Figure 3:**
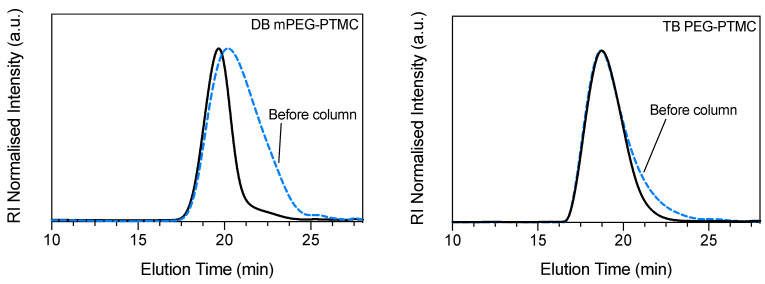
GPC traces for DB mPEG-PTMC (**left**) and TB PEG-PTMC (**right**) before (dashed-blue line) and after (solid black line) column purification. For the DB copolymer a satisfactory separation during flash chromatography could not be obtained and, thus, a relatively significant loss of low molecular weight mPEG-PTMC chains was unavoidable.

An optimal separation could not be obtained for the DB copolymer mPEG-PTMC, regardless of the used conditions (e.g., different eluents, concentration etc), which led to a fairly significant loss of lower molecular weight species (Figure 3, left). In turn, this caused a substantial shift towards higher *M_n_^GPC^* and *M_p_^GPC^* values, as well as a drastically augmented hydrophobic/hydrophilic repeat unit ratio (*R*), for this copolymer upon column chromatography (Table 4). Conversely, the shifts in *M_p_^GPC^* and *R* ratio were considerably less significant for the TB PEG-PTMC copolymer. Expectedly, the removal of lower molecular species resulted in a diminished dispersity (i.e., narrower molecular weight distribution) for both polymers after this purification process. In particular, for the DB mPEG-PTMC, despite the presence of a relatively long tail still attributable to some residual low molecular weight chains (approximately from 21 to 24 min), a relatively narrow dispersity of 1.3 was detected upon column chromatography.

The chromatographic purification process was monitored by performing TLC analyses on the eluted fractions, and the characteristics of the discarded species were determined via ^1^H-NMR studies. While in the case of the TB PEG-PTMC copolymer NMR spectroscopy confirmed the exclusive removal of PTMC homopolymer upon silica gel chromatography, for the DB mPEG-PTMC the discarded fractions were composed of a mixture of PTMC homopolymer and lower molecular weight mPEG-PTMC species, as expected from the poor separation highlighted via TLC. For increased clarity, the ^1^H-NMR spectra of the discarded side-products are shown in the SI, together with a selection of photos of the TLC plates.

The structural and thermal characteristics of the copolymers, before and after the column purification process, together with those of the synthesised PTMC homopolymer, are reported in Table 4. Interestingly, the column chromatography purification had merely minor effects on the thermal properties of both copolymers, resulting only in a slight shift of the *T_g_* towards higher temperatures. Whilst a thorough investigation on the reasons behind this observation is beyond the scope of this study, it can be postulated that the two copolymers were most likely above their respective critical entanglement molecular weights already before the chromatographic purification and, hence, the molecular weight variation did not have a noteworthy impact on their specific thermal characteristics [46]. Additionally, while the shift in *M_n_* detected by GPC after column purification was significant for both polymers, the change in *M_p_^GPC^* was relatively less important for the DB mPEG-PTMC and nearly non-existent for the TB copolymer.

Despite the limited separation obtained during the purification of the DB copolymer, and the resulting substantial alterations in *M_n_* and hydrophobic/hydrophilic units ratio described above, silica gel flash chromatography allowed for the obtainment of a purer DB polymer with characteristics suitable for the in vivo degradation studies. Therefore, the two products purified via column chromatography were used for the in vivo experiments in order to highlight and distinguish more clearly the behaviour associated to purer TB PEG-PTMC, DB mPEG-PTMC and PTMC homopolymer. This allowed to exclude any possible contribution attributable to the presence of PTMC by-product in the copolymers, thus ensuring a more precise structure-property-degradation assessment.

### 3.3. In Vivo Degradation Studies

The PTMC homopolymer and the two amphiphilic copolymers, purified through silica gel column chromatography, were injected in the flank of Sprague Dawley rats to assess their degradation behaviour in vivo over three months and discern any differences attributable to the presence of the hydrophilic PEG block in the two copolymers.

**Figure 4:**
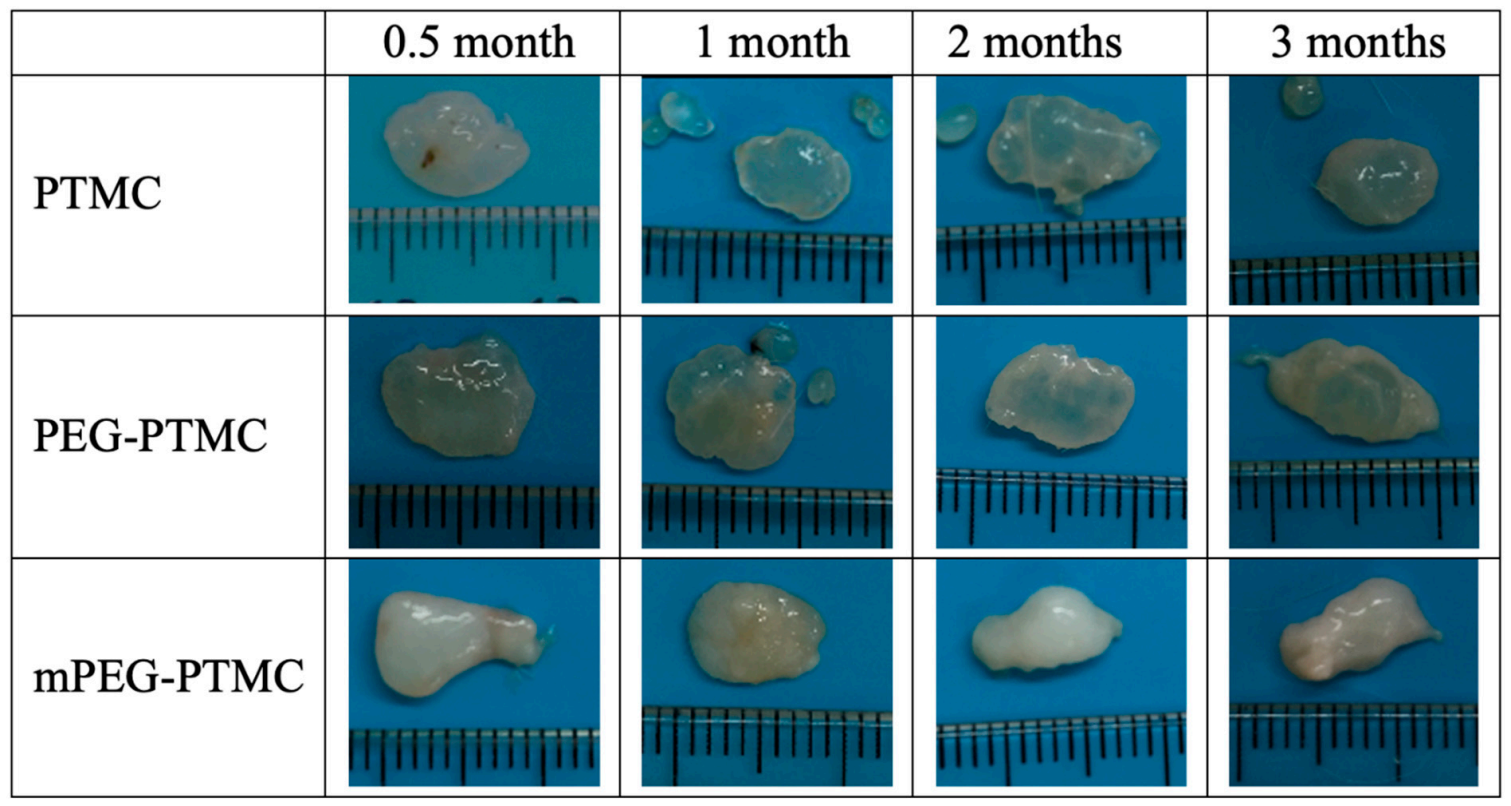
PTMC-based polymer depots after excision at different times (0.5, 1, 2 and 3 months). Each of the tick marks on the ruler represents 1 mm.

After explantation at specific time points (0.5 month, 1 month, 2 months and 3 months), the polymer depots were carefully analysed. The visual aspect of a selection of the explanted depots can be seen in Figure 4. Most of the depots were mainly transparent/translucent, in agreement with the amorphous nature of the pristine polymers. However, some more white/opaque regions were also visible for some depots (in particular for those of the DB mPEG-PTMC) and could likely be due to the interaction of the polymers with water and biological fluids at the surface of the structures, as observed elsewhere for other PTMC-based implants [36].

For all the polymer depots no univocal or significant changes in dimensions and visual appearance were observed during this 3-month degradation study; expectedly confirming a relatively slow degradation progression mainly due to a surface erosion process [31,36,38,39]. In a similar manner, the structural characteristics determined by GPC displayed that the decrease in molecular weight was relatively not significant over the course of this study (Table 5).

**Figure 5:**
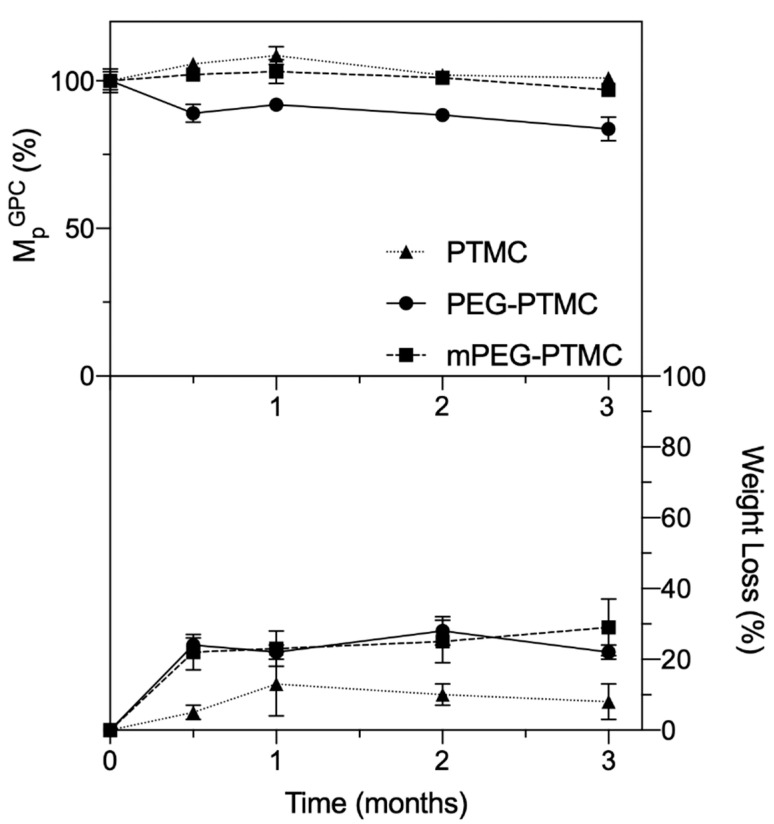
Relative M_p_^GPC^ (**top**) and weight loss change (**bottom**) over time for the three polymers during in vivo studies in rats. As expected, no significant changes in molar mass where detected due to the degradation proceeding via surface erosion. The experiments were performed in quadruplicates (four different depots from different animals analysed at each time point). The lines connective the symbols are included as a guide to the eye.

To mitigate any source of variability and errors resulting from the small, but nonetheless detected, changes in dispersity that could influence the trends of *M_n_^GPC^* during the study, the *M_p_^GPC^* values were plotted and evaluated as a function of time (Figure 5, top). Expectedly, this value was fairly stable over time due to the degradation proceeding by surface erosion, as already observed by Zhang et al. for other PTMC homopolymers in a study with comparable duration (up to 20 weeks) [38].

In our case, a slightly more pronounced degradation was observed for the TB PEG-PTMC. This was further confirmed by weight loss analyses (Figure 5, bottom). In fact, by looking at the gravimetric data, during the course of this study the two amphiphilic copolymers degraded comparatively more than the PTMC homopolymer. In more detail, the two copolymers displayed a 20–30% weight loss already after 2 weeks, while for the homopolymer a decrease in weight of less than 10% was observed at the same time point. At later stages, the weight loss was somewhat stable for all the polymers, and at the final time point (3 months) the residual weight was still around 70–80% of the starting value for the copolymers, and ~90% for the PTMC homopolymer. Additionally, the fact that the detected weight loss was somewhat faster compared to the molecular weight decrease further confirmed that the degradation was fundamentally due to a process of surface erosion [36,38].

Analogous trends have been observed elsewhere when comparing several homopolymers of PTMC characterised by different molar masses [31,38]. In these cases, the higher molecular weight polymers degraded significantly faster and this was attributed to a different conformation of the adsorbed enzymes on the surface of polymers with higher molar mass which possessed different surface characteristics that, in turn, resulted in a faster degradation [31].

In our systems, rather than to the molar mass itself, this difference might presumably be attributed to the necessarily different surface properties and chain arrangements of the amphiphilic copolymers compared to those of the PTMC homopolymer. These structural and chemical differences might likely have an impact on the extent of degradation by affecting the conformation of the adsorbed enzymes on the surface of the polymers and, hence, ultimately altering the degradation activity of the biological catalysts. Further studies, beyond the scope of this paper, might be useful to completely comprehend this behaviour and fully unveil the influence of the presence of the PEG block on the activity of the enzymes responsible for the degradation of the PTMC segments in PEG-PTMC copolymers. For instance, investigations of this type have already been done via atomic force microscopy (AFM) by other groups on polyesters and other PTMC homopolymers [43,47] and might help in understanding the reasons behind this observed behaviour by enabling for the visualization of the biocatalysts arrangement on the polymer surface at the nanometre scale.

Finally, the generally limited extent and rate of degradation displayed by the three polymers might be rationalised and explained in terms of their low molecular weight. Indeed, it is well known and widely reported in literature that the rate of mass loss of high molecular weight PTMC specimens is substantially higher than that observed for lower molecular weight analogues [36,43]. Therefore, the use of PTMC-based polymers with a relatively low molecular weight, like the ones used in this study, might specifically be of particular interests for all those LAI applications where somewhat long degradation and resorption times are desired.

### 3.4. Acylation and Drug Recovery Studies

As described in the introduction, the utilisation of polyesters as excipients for the formulation of LAIs and for ISFDs might potentially introduce several drawbacks, such as the generation of acidic degradation products, possible high water-intake, potential instability of some therapeutic entities due to acylation, etc. [23,24].

In particular, in formulations including PLAs and/or PLGAs, nucleophilic moieties such as the primary amino groups have been shown to be the major targets for acylation, as a result of their interaction with lactic and glycolic acid units resulting from the scission of the ester linkages within the polymer backbone [24].

Therefore, the utilisation of aliphatic polycarbonate-based amphiphatic copolymers (i.e., PEG-PTMCs), as alternatives to PEG-polyesters, can provide the solution to mitigate or suppress all the issues associated to the presence of acidic degradation by-products and the consequent acylation of labile APIs.

Octreotide acetate and liothyronine are pharmaceutical ingredients containing amino groups known to undergo acylation [24,25,43]. In order to assess the applicability of PTMC-based amphiphilic block copolymers for the delivery of these two sensitive APIs, drug recovery studies were performed by using the synthesised TB PEG-PTMC and comparing it to a purchased TB PEG-PLA with analogous structural features (i.e., comparable molar mass and hydrophobic/hydrophilic repeat units ratio).

**Figure 6:**
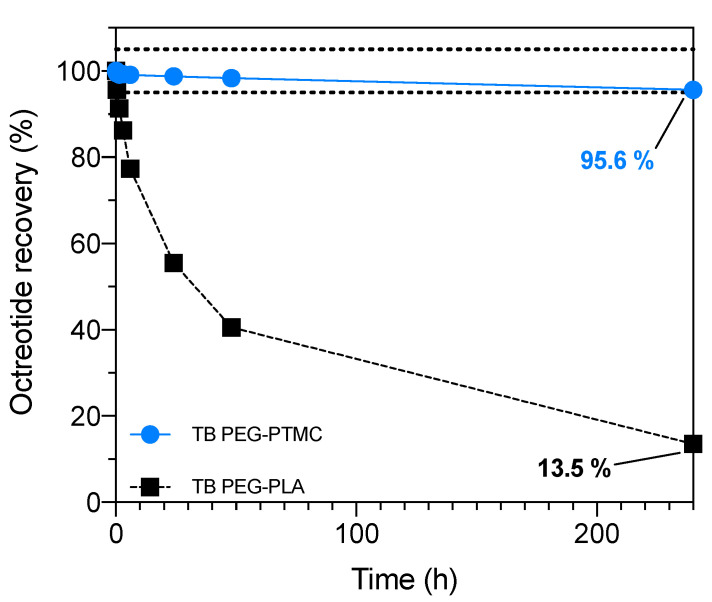
Octreotide recovery over time quantified through HPLC in formulations containing: TB PEG-PTMC (blue) and TB PEG-PLA (black). At the end of the study, more than 95% of the API was retrieved when PEG-PTMC was used. The lines connecting the symbols are included as a guide to the eye. The error was typically less than 1% and, thus, the data point symbols are bigger than the error bars. The drug recovery area between 100 ± 5% is highlighted within the two dotted lines.

Drug recovery studies performed through HPLC and UPLC experiments lucidly confirmed that, whilst in the presence of PEG-PLA both octreotide and liothyronine were rapidly negatively affected, when formulated with the TB PEG-PTMC copolymer the recovery was always extremely high. In more detail, for octreotide more than 95% of the API was recovered in the presence of the PTMC-based TB block copolymer even after 10 days. Conversely, when utilising PEG-PLA, 60% of the active ingredient was degraded already after 40 h and the drug recovery at the final time point (10 days) was as low as ~14% (Figure 6).

**Figure 7:**
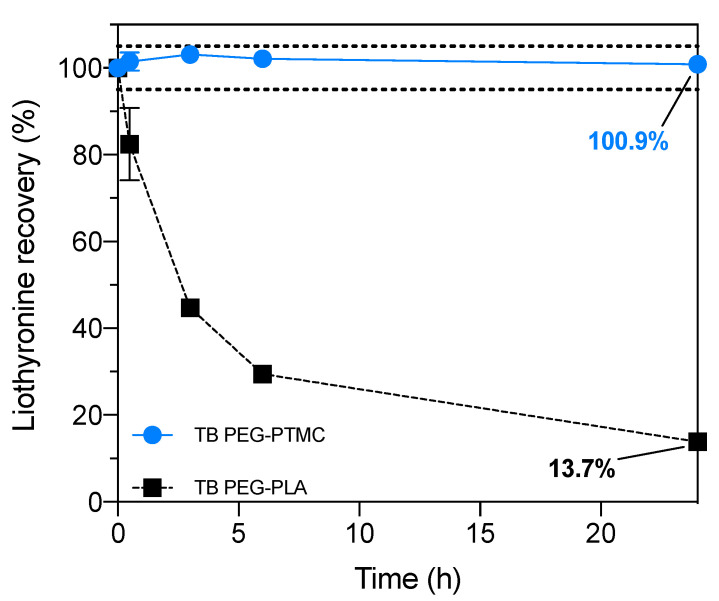
Liothyronine recovery over time quantified through UPLC in formulations containing: TB PEG-PTMC (blue) and TB PEG-PLA (black). At the end of the study, around 100% of the API was retrieved when PEG-PTMC was used. The lines connecting the symbols are included as a guide to the eye. The error was typically less than 1% and, thus, the data point symbols are bigger than the error bars. The drug recovery area between 100 ± 5% is highlighted within the two dotted lines.

In a similar fashion, in the case of liothyronine, which is a relatively unstable and sensitive thyroid hormone used to treat hypothyroidism and myxedema coma [42,48], when using PEG-PLA the recovery was already merely ~30% after 6 h, and around 14% after 24 h. On the other hand, with PEG-PTMC the recovery was quantitative (~100%) all over the time span of the study (Figure 7), thus confirming the usefulness and added value of PTMC-based amphiphilic block copolymers as bioresorbable materials for the sustained release of APIs containing chemical groups susceptible to acylation.

## 4. Conclusions

Two (m)PEG-PTMC copolymers and one PTMC homopolymer were successfully synthesised and purified to assess and compare their degradation behaviour in vivo, with the final aim of evaluating them as resorbable materials for the formulation of LAIs.

The 3-month in vivo degradation study of the three PTMC-based polymers evidently displayed characteristics associated with a surface erosion process. Accordingly, the rate of weight loss for all the specimens was substantially higher than their respective molecular weight decrease.

A more significant weight loss was detected for the two amphiphilic copolymers, which might likely be attributed to the different surface properties and to the arrangement of the entangled chains that influence the adsorption of the degrading enzymes on the polymeric surface.

Finally, with regard to the acylation issues frequently encountered when formulating peptides and other APIs containing amino groups with PEG-polyesters, both octreotide acetate and liothyronine sodium were remarkably stable in the presence of TB PEG-PTMC. Conversely, a significantly quicker and more substantial acylation was observed for the same APIs when using formulations containing an analogous TB PEG-PLA.

In conclusion, (m)PEG-PTMC copolymers have been proven to be viable candidates for the preparation of resorbable materials with strong potential as constituents for ISFD systems and, in particular, for all those LAI applications where a rather long drug delivery is desired. Additionally, the results of our study unambiguously demonstrate the usefulness and advantages afforded by using PTMC-based copolymers as bioresorbable implants for the sustained delivery of active molecules typically prone to acylation.

## Figures and Tables

**Table 1:** Naming scheme used for the polymers presented in this study.

Polymer	Type	Abbreviation
Methoxy poly(ethylene glycol)-*b*-poly(1,3-trimethylene carbonate)	Diblock (DB)	mPEG-PTMC
Poly(1,3-trimethylene carbonate)-*b*-poly(ethylene glycol)-*b*-poly(1,3-trimethylene carbonate)	Triblock (TB)	PEG-PTMC
Poly(1,3-trimethylene carbonate)	Homopolymer	PTMC

**Table 2:** Composition of the formulations injected in the in vivo studies.

Formulation	Solvent	Polymer (Type)	Concentration (wt.%)
(a)	DMSO	PEG-PTMC (TB)	40
(b)	DMSO	mPEG-PTMC (DB)	40
(c)	DMSO	PTMC (homopolymer)	40

**Table 3:** Composition of the formulations used for the acylation studies. DMSO was used as a solvent for all the formulations.

Formulation	API (wt.%)	Polymer (wt.%)
1	Octreotide (4%)	PEG-PTMC (35%)
2	Octreotide (4%)	PEG-PLA (35%)
3	Liothyronine (1%)	PEG-PTMC (35%)
4	Liothyronine (1%)	PEG-PLA (35%)

**Table 4:** Molecular weight analyses and thermal attributes of the copolymers presented in this study. The characteristics before and after column purification are shown for the two amphiphilic copolymers.

Product	*M_n_^GPC^* (kg mol^−1^) ^a^	*Ð* ^a^	*M_p_^GPC^* (kg mol^−1^) ^a^	*M_n_^NMR^* (kg mol^−1^) ^b^	*R* ^b^	*T_g_* (°C) ^c^
PTMC	6.0	1.7	10.5	5.8	-	−27
PEG-PTMC	7.9	2.1	17.7	10.1	3.9	−30
PEG-PTMC (after column)	12.7	1.8	17.2	10.5	4.1	−28
mPEG-PTMC	3.3	2.2	7.9	4.9	5.6	−29
mPEG-PTMC (after column)	7.9	1.3	9.6	7.0	7.3	−26

^a^ Determined through GPC-TDA in THF (15 mg mL^−1^, 1 mL min^−1^, 30 °C), molecular weight values rounded to the closest hundred. ^b^ Determined through ^1^H-NMR in CDCl_3_ (15–20 mg mL^−1^), molecular weight values rounded to the closest hundred. ^c^ Determined through DSC from the second heating scan (10 °C min^−1^, N_2_), values rounded to the closest integer. GPC and DSC studies were performed at least in duplicates, see experimental section for error estimates.

**Table 5:** GPC-TDA data at t_0_ and at final data point (3 months) after injection in rats. No substantial changes were detected at the two extremes of the timespan of this study.

Sample	Time Point (Month)	*M_n_^GPC^* (kg mol^-1^) ^a^	*Đ* ^a^	*M_p_^GPC^* (kg mol^-1^) ^a^
PTMC	0	6.0	1.7	10.5
	3	5.8	1.8	10.7
PEG-PTMC	0	12.7	1.8	17.2
	3	10.3	1.5	14.4
mPEG-PTMC	0	7.9	1.3	9.6
	3	7.0	1.5	9.3

^a^ Determined through GPC-TDA in THF (15 mg mL^−1^, 1 mL min^−1^, 30 °C), molecular weight values rounded to the closest hundred. Experiments performed in quadruplicates on different depots explanted at the same point, see experimental section for GPC error estimates.

## Data Availability

The data presented in this study are available on request from the corresponding author.

## Supplementary Materials

The SI are available online at. Table S1: HPLC method for the quantification of octreotide acetate. Table S2: UPLC method for the quantification of liothyronine. Figure S1: ^1^H-NMR spectrum of diblock mPEG-PTMC in CDCl_3_. The integrals were normalised to the resonance assigned to the methoxyl protons (3.38 ppm). A minor amount of acetone residue is detected at 2.16 ppm (0.3 wt.%). The peak at 4.45 ppm is attributed to residual unreacted TMC monomer. Figure S2: ^1^H-NMR spectrum of triblock PEG-PTMC in CDCl_3_. The integrals were normalised to the resonance assigned to the PEG 1000 used as initiator (3.8-3.6 ppm). A minor amount of acetone residue is detected at 2.16 ppm (0.2 wt.%). The peak at 4.45 ppm is attributed to residual unreacted TMC monomer. Figure S3: ^1^H-NMR spectrum of PTMC homopolymer in CDCl_3_. The integrals were normalised to the resonance assigned to the terminal -CH_3_ of the octanol initiator (0.88 ppm). Minor amounts of acetone (2.16 ppm, 0.4 wt.%) and water (1.56 ppm, 0.6 wt.%). The peak at 4.45 ppm is attributed to residual unreacted TMC monomer. The end-group aliphatic protons of the PTMC chain (-CH_2_-OH) are detected at around 3.75 ppm. Figure S4: TLC plates obtained for the triblock PEG-PTMC: crude product (left), product after precipitation (centre), and polymer after column chromatography (right). Separate and well-defined spots could be obtained in all cases. Figure S5: TLC plates obtained for the diblock mPEG-PTMC: product before column purification (left), and polymer after column chromatography (right). In this case, a clear separation was not obtained, regardless of the conditions used (e.g. eluents, concentration etc.), and the two spots were connected by a streak. Figure S6: ^1^H-NMR spectrum in CDCl_3_ of the by-product removed from the triblock copolymer PEG-PTMC. The characteristic resonances attributed to the protons of PTMC (4.23 ppm and 2.03 ppm) could exclusively be detected. No clear signals were observed in the characteristic area associated with the chemical groups of PEG 1000 (area highlighted with a dashed-red box for clarity). Figure S7: ^1^H-NMR spectrum in CDCl_3_ of the initial fractions (side-product) obtained from the purification of the diblock copolymer mPEG-PTMC. As a consequence of the impossibility of obtaining a good separation during the chromatographic purification, the discarded products contained PTMC homopolymer and mPEG-PTMC species. In addition to the PTMC signals (4.23 ppm and 2.03 ppm), the characteristic peaks associated to the presence of the mPEG 350 block are visible at around 3.8-3.4 ppm (area highlighted with a dashed-red box for clarity).

## Sample Availability

Available from the authors.
