# Supplementary material for: Poly(ethylene glycol)-b-poly(1,3-trimethylene carbonate) Amphiphilic Copolymers for Long-Acting Injectables: Synthesis, Non-Acylating Performance and In Vivo Degradation
Source: Molecules. 2021 Mar 6;26(5):1438. doi: 10.3390/molecules26051438 (PMC7962012; doi:10.3390/molecules26051438)
Supplement: Supplementary file 1 [file molecules-26-01438-s001.pdf]

## SUPPORTING INFORMATION

**Poly(ethylene glycol)-*b*-poly(1,3-trimethylene carbonate) amphiphilic copolymers for long- acting injectables: synthesis, non-acylating performance and *in vivo* degradation**

Silvio Curia, Feifei Ng, Marie-Emérentienne Cagnon, Victor Nicoulin, Adolfo Lopez-Noriega
*MedinCell, 3 Rue des Frères Lumière, Jacou 34830, France*

Pages = 8 (including cover sheet)

Tables = 2

Figures = 7

*1. HPLC and UPLC methods utilised for the drug recovery studies*

The following methods were used to quantify the amount of API (octreotide or liothyronine) at each time point following the acylation studies.

*Table 1* - HPLC method for the quantification of octreotide acetate.

| | |
|---|---|
| Column | Aeris WIDERPORE XB-C18; (3.6 µm, 200 Å, 150 x 4.6 mm), Phenomenex |
| Security guard | SecurityGuard ULTRA Holder for UHPLC Columns, 2.1 to 4.6 mm ID, Ea |
| Column temperature | 40 °C |
| Mobile phase A | Acetonitrile + 0.1% TFA |
| Mobile phase B | $H_2O$ + 0.1% TFA |
| Volume injected | 10 µL |
| Flow rate | 1 mL min$^{-1}$ |
| Retention time | 5.30 min |
| Wavelength | 222 nm |
| Gradient | |

| Time (min) | %A | %B |
|---|---|---|
| **0** | 15 | 85 |
| **0.1** | 15 | 85 |
| **8** | 45 | 55 |
| **9** | 90 | 10 |
| **10** | 90 | 10 |
| **11.5** | 15 | 85 |
| **17** | 15 | 85 |

*Table 2 - UPLC method for the quantification of liothyronine.*

| | |
|---|---|
| Column | Acquity UPLC BECH C18 column (1.7 µm, 130 Å, 2.1 x 50 mm) |
| Pre-column | Acquity Col.In-Line filters 0.50 mm |
| Column temperature | 35 °C |
| Mobile phase A | Acetonitrile + 0.1% Formic Acid |
| Mobile phase B | $H_2O$ + 0.1% Formic Acid |
| Volume injected | 1 µL |
| Flow rate | 0.6 mL $min^{-1}$ |
| Retention time | 3.36 min |
| Wavelength | 297 nm |
| Gradient | |

| Time (min) | %A | %B |
|---|---|---|
| **0** | 5 | 95 |
| **8.0** | 95 | 5 |
| **12.0** | 95 | 5 |
| **12.5** | 5 | 95 |
| **15** | 5 | 95 |

## 2. *$^{1}$H-NMR spectra of the polymer products*

$^1$H-NMR studies in $CDCl_3$ showed successful synthesis of the expected polyesters. The spectra of the two copolymers and that of the PTMC homopolymer are shown below (Figure 1S to Figure 3S). All the expected resonances could be unambiguously detected.

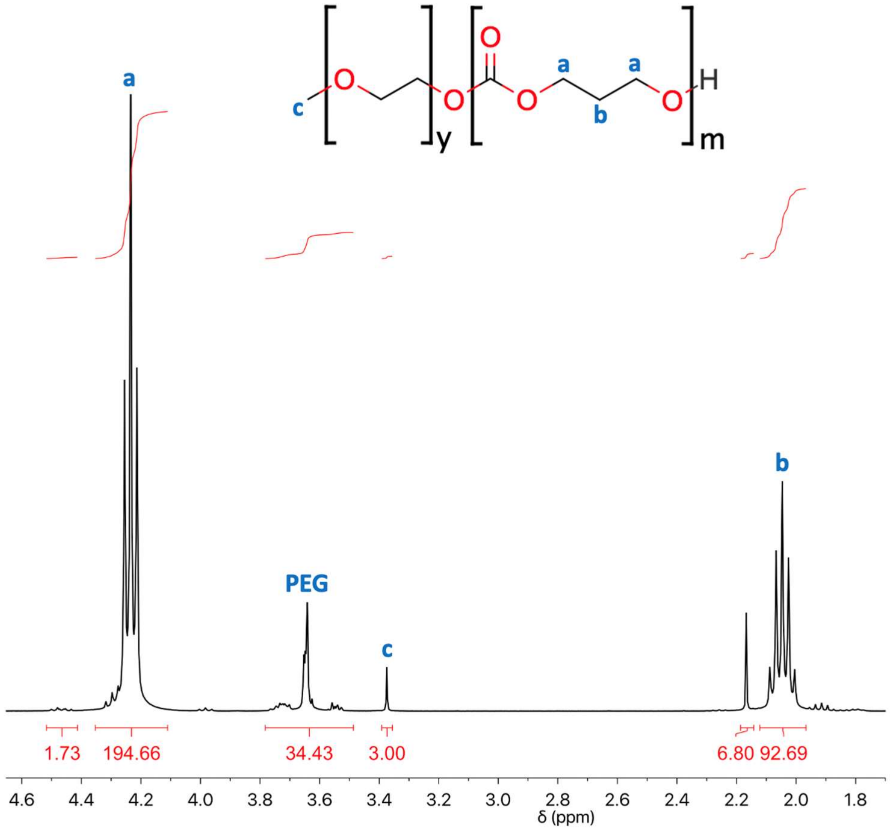


*Figure 1S- $^1$H-NMR spectrum of diblock mPEG-PTMC in $CDCl_3$. The integrals were normalised to the resonance assigned to the methoxyl protons (3.38 ppm). A minor amount of acetone residue is detected at 2.16 ppm (0.3 wt.%). The peak at 4.45 ppm is attributed to residual unreacted TMC monomer.*

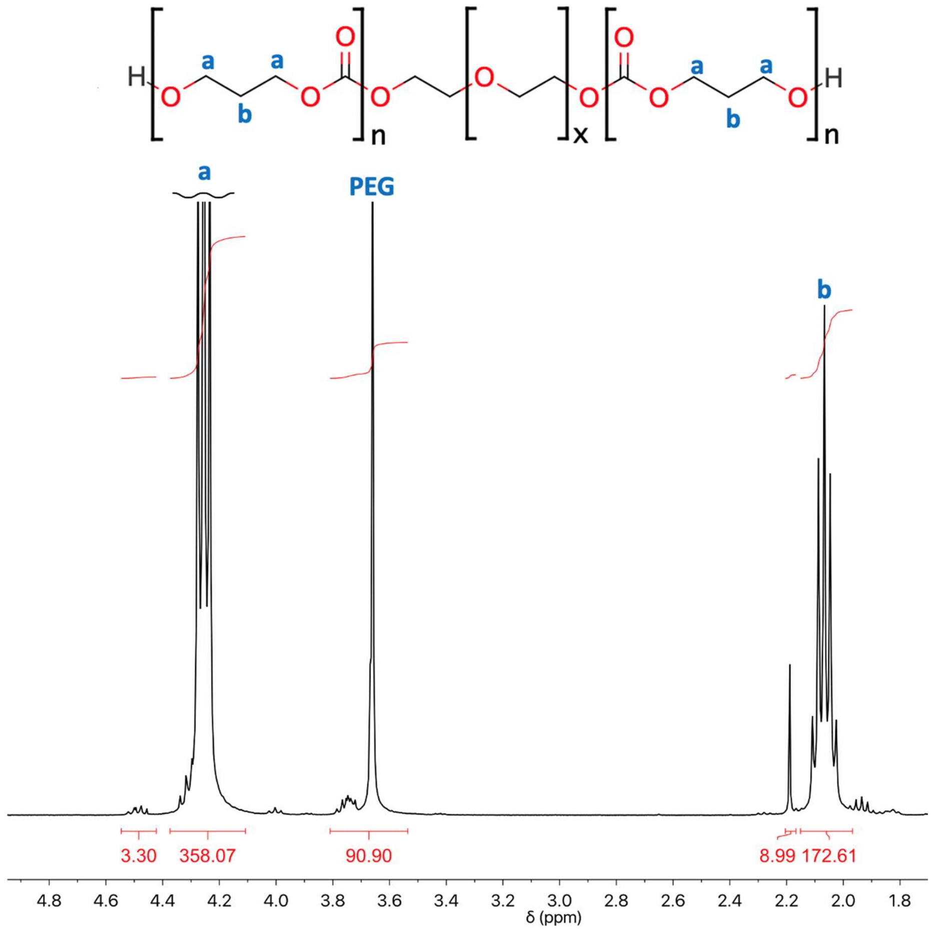


*Figure 2S - $^{1}$H-NMR spectrum of triblock PEG-PTMC in $CDCl_3$. The integrals were normalised to the resonance assigned to the PEG 1000 used as initiator (3.8-3.6 ppm). A minor amount of acetone residue is detected at 2.16 ppm (0.2 wt.%). The peak at 4.45 ppm is attributed to residual unreacted TMC monomer.*

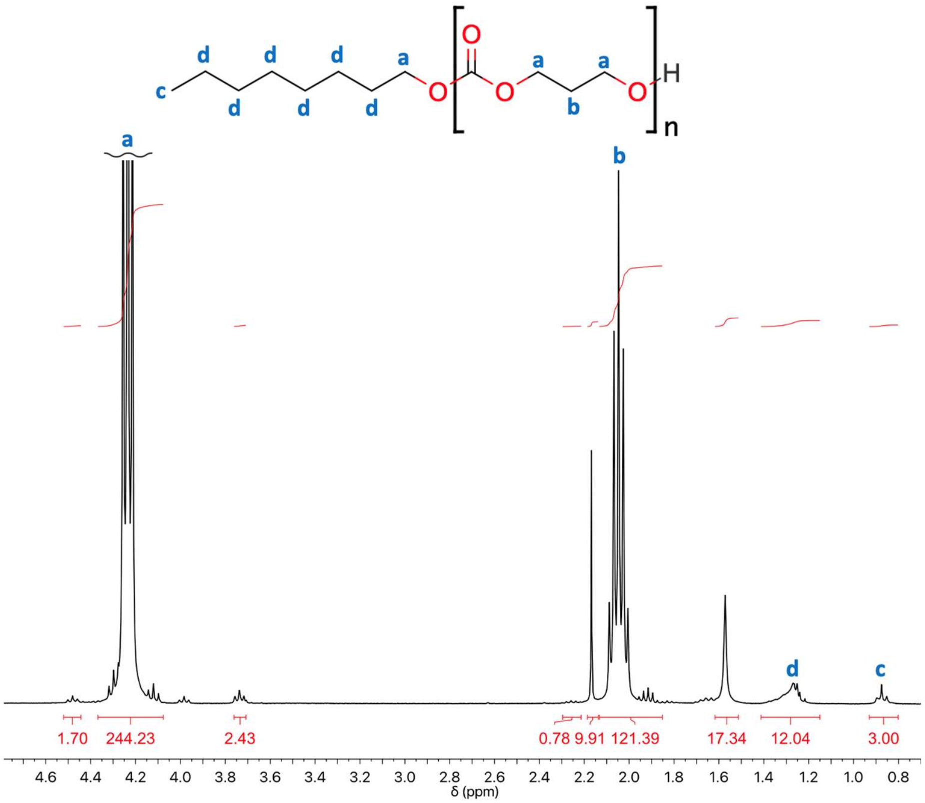


*Figure 3S - $^{1}$H-NMR spectrum of PTMC homopolymer in $CDCl_3$. The integrals were normalised to the resonance assigned to the terminal $-CH_3$ of the octanol initiator (0.88 ppm). Minor amounts of acetone (2.16 ppm, 0.4 wt.%) and water (1.56 ppm, 0.6 wt.%). The peak at 4.45 ppm is attributed to residual unreacted TMC monomer. The end-group aliphatic protons of the PTMC chain ($-CH_2-OH$) are detected at around 3.75 ppm.*

*3. TLC analyses*

As described in the manuscript, the use of column chromatography allowed for the purification of the copolymers to eliminate any possible contribution attributable to the presence of PTMC by-product during the *in-vivo* studies, ultimately ensuring a more precise structure-property-degradation evaluation.

TLC was used to monitor the whole process and analyse the content of the separated fractions. While the separation obtained for the triblock PEG-PTMC was extremely satisfactory (Figure 4S), an optimal separation could not be obtained for the diblock copolymer mPEG-PTMC (Figure 5S).

The TLC plate of the crude product is also shown for the triblock PEG-PTMC in Figure 4S, to visually highlight the efficacy of the complete purification process (*i.e.* precipitation in EtOH to remove low molecular weight species and monomer residues followed by column chromatography to remove the PTMC homopolymer by-product).

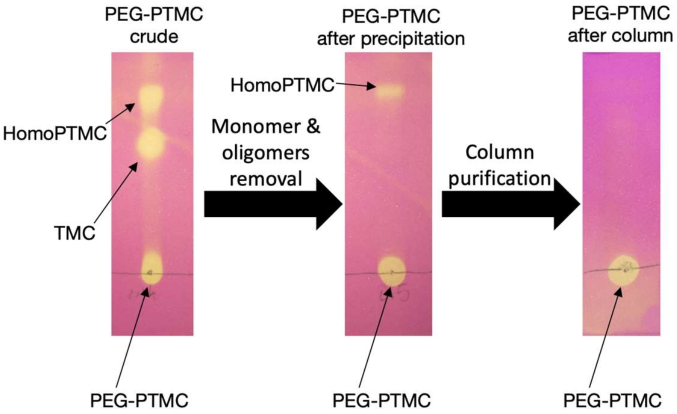


*Figure 4S – TLC plates obtained for the triblock PEG-PTMC: crude product (left), product after precipitation (centre), and polymer after column chromatography (right). Separate and well-defined spots could be obtained in all cases.*

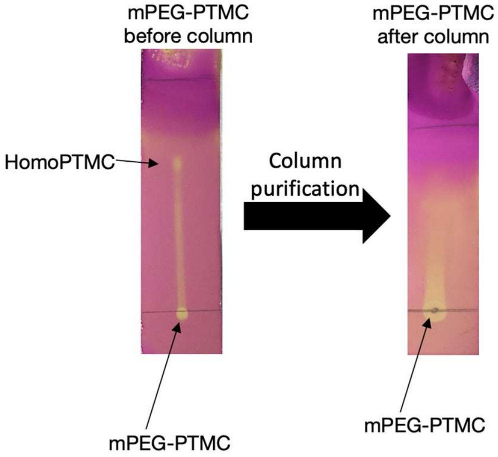


*Figure 5S - TLC plates obtained for the diblock mPEG-PTMC: product before column purification (left), and polymer after column chromatography (right). In this case, a clear separation was not obtained, regardless of the conditions used (e.g. eluents, concentration etc.), and the two spots were connected by a streak.*

## 4. $^{1}$H-NMR spectra of fractions discarded upon column chromatography

$^{1}$H-NMR studies performed on the fractions discarded upon column chromatography confirmed that the side-product eliminated for the triblock PEG-PTMC was composed only of PTMC homopolymer. As a matter of fact, only the resonances attributed the chemical groups of PTMC groups were detected (Figure 6S).

On the other hand, the spectrum collected on the fractions discarded from the purification of the diblock mPEG-PTMC presented both the characteristic signals attributed to the protons of PTMC and those of mPEG 350 (Figure 7S). Therefore, in the case of the diblock copolymer, due to the poor separation during chromatography, the discarded fractions were composed of a mixture of PTMC homopolymer and mPEG-PTMC chains. Accordingly, while for the TB copolymer the amount of by-product removed after purification (composed only of PTMC homopolymer) accounted for 13% of the initial sample weight, for the DB mPEG-PTMC around 52% of the initial mass was discarded upon column purification, but this larger quantity included also copolymer chains.

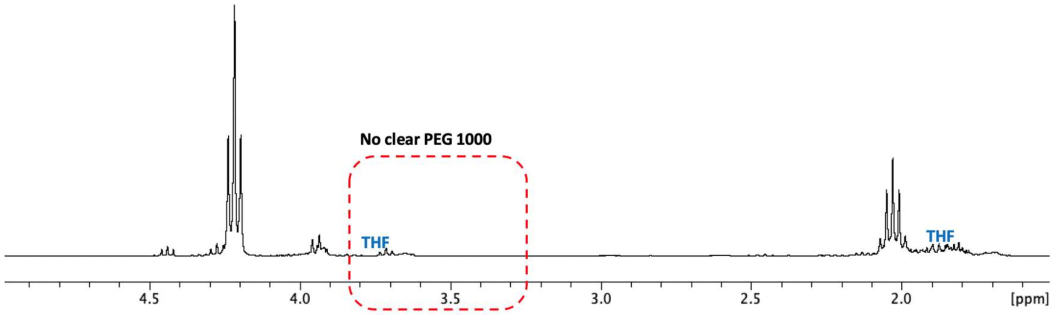


*Figure 6S - $^{1}$H-NMR spectrum in $CDCl_3$ of the by-product removed from the triblock copolymer PEG-PTMC. The characteristic resonances attributed to the protons of PTMC (4.23 ppm and 2.03 ppm) could exclusively be detected. No clear signals were observed in the characteristic area associated with the chemical groups of PEG 1000 (area highlighted with a dashed-red box for clarity).*

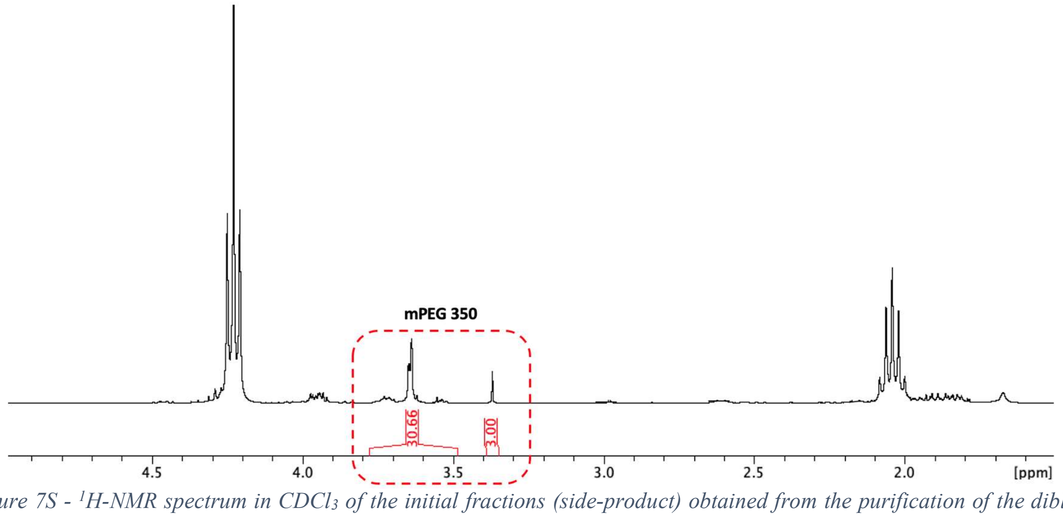


*Figure 7S - $^{1}$H-NMR spectrum in $CDCl_3$ of the initial fractions (side-product) obtained from the purification of the diblock copolymer mPEG-PTMC. As a consequence of the impossibility of obtaining a good separation during the chromatographic purification, the discarded products contained PTMC homopolymer and mPEG-PTMC species. In addition to the PTMC signals (4.23 ppm and 2.03 ppm), the characteristic peaks associated to the presence of the mPEG 350 block are visible at around 3.8-3.4 ppm (area highlighted with a dashed-red box for clarity).*
